# Acupuncture for Obstructive Sleep Apnea (OSA) in Adults: A Systematic Review and Meta-Analysis

**DOI:** 10.1155/2020/6972327

**Published:** 2020-03-05

**Authors:** Liaoyao Wang, Jia Xu, Yijun Zhan, Jian Pei

**Affiliations:** ^1^Department of Acupuncture, Longhua Hospital, Shanghai University of Traditional Chinese Medicine, Shanghai 200032, China; ^2^Department of Acupuncture, Special Wards, Longhua Hospital, Shanghai University of Traditional Chinese Medicine, Shanghai 200032, China

## Abstract

**Objective:**

Our aim was to assess the efficacy and safety of acupuncture for OSA patients with various severities of the disorder.

**Methods:**

Eight databases including PubMed, Cochrane Library, EMBASE, Web of Science, China National Knowledge Infrastructure (CNKI), Chongqing VIP (CQVIP), Wanfang Data, and Chinese Biomedical Literature Database (CBM) were comprehensively searched till July 2019. Randomized controlled trials (RCTs) testing acupuncture in the treatment of OSA were eligible for inclusion. Studies were selected for inclusion, and data were extracted by two authors independently. The Cochrane Collaboration's Risk of Bias Assessment Tool and RevMan software (version 5.3) were used to evaluate the quality of studies and conduct statistical analysis.

**Results:**

Nine RCTs with 584 participants were included. The trials covered acupuncture and electropuncture. Acupuncture caused clinically significant reductions in AHI (MD: -6.18; 95% CI: -9.58 to -2.78; *Z* = 3.56, *P* = 0.0004) as well as in ESS (MD: -2.84; 95% CI: -4.80 to -0.16, *Z* = 2.09, *P* = 0.04). AHI was reduced more in the subgroup analysis of moderate OSA patients (MD: -9.44; 95% CI: -12.44 to -6.45; *Z* = 6.18, *P* < 0.00001) and severe OSA patients (MD: -10.09; 95% CI: -12.47 to -7.71; *Z* = 8.31, *P* < 0.00001). ESS was also reduced more in the subgroup analysis of moderate OSA patients (MD: -2.40; 95% CI: -3.63 to -1.17; *Z* = 3.83, *P* = 0.0001) and severe OSA patients (MD: -4.64; 95% CI: -5.35 to -3.92; *Z* = 12.72, *P* < 0.00001). Besides, acupuncture had a beneficial effect on LSaO_2_ (MD: 5.29; 95% CI: 2.61 to 7.97; *Z* = 3.86, *P* = 0.0001). The outcome of AHI and LSaO_2_ yielded consistent results after sensitivity analysis, but the direction of the outcome of ESS was reversed. And the quality of evidence was mainly low to very low.

**Conclusions:**

Acupuncture therapy is effective for OSA patients in reducing AHI and ESS and in improving the LSaO_2_ of various severities, especially in moderate and severe OSA patients. High-quality trials are urgently needed.

## 1. Introduction

Obstructive sleep apnea (OSA) is a common sleep disorder causing excessive daytime sleepiness, increasing risk for motor vehicle accidents, and impairing quality of life, and it is closely associated with cardiovascular events, diabetes, and other diseases [1–4]. Approximately 936 million adults worldwide have mild to severe OSA and 425 million have moderate to severe OSA; the prevalence exceeds 50% in some countries, and China is the most affected [5]. OSA is associated with high economic and social burdens—the cost for diagnosis and treatment in the United States was up to $12.4 billion in 2015 [6]. Because of the high prevalence, hazard, and economic burden, the prevention and treatment of OSA is of great importance. Currently, therapies for OSA have been designed and proposed to reduce the frequency of sleep-disordered breathing events, including continuous positive airway pressure (CPAP) [7], surgical procedures [8], weight loss and lifestyle interventions [9, 10], and pharmacotherapy [11]. One traditional and novel intervention is the application of acupuncture [12].

Previous meta-analysis of acupuncture for OSA including 6 randomized controlled trials (RCTs) involving 362 patients was done by Lv et al. [13] and was published in 2016. The review [13] showed that acupuncture was more effective in improving the apnea-hypopnea index (AHI) and further improved the apnea index and hypopnea index. However, the review can only demonstrate the overall efficacy of acupuncture on OSA. Subgroup analysis of OSA severity was not performed, and the efficacy of acupuncture may vary from severity. Furthermore, several relevant studies on the efficacy of acupuncture for treating OSA have been published since 2016. Thus, we undertook a systematic review and meta-analysis of RCTs to evaluate the efficacy and safety of acupuncture for OSA according to the severity of the disease.

## 2. Methods

Our review was performed strictly with the recommendations of the PRISMA (Preferred Reporting Items for Systematic Reviews and Meta-Analyses statement) [14] guidelines.

### 2.1. Eligibility Criteria

Only RCTs were included in this review, and they had to meet all of the following criteria: (1) were performed in OSA adult patients with different grades of severity in accordance with AHI (individuals with AHI values of 5~15, 16~30, or more than 30 times per hour were considered to have mild, moderate, or severe OSA, respectively) [2, 15]; (2) assessed the effects of acupuncture (electroacupuncture, manual acupuncture, etc.) compared with a control group (sham acupuncture or CPAP or pharmacotherapy or receiving no treatment); and (3) included at least one of the following outcomes: apnea-hypopnea index (AHI), Epworth Sleepiness Score (ESS), lowest oxygen saturation (LSaO_2_), and adverse effect.

Studies were excluded if they met the following criteria: (1) OSA as a complication; (2) repeated publications (data were only extracted from one study that was recently updated); and (3) acupuncture treatment was performed less than 7 times.

### 2.2. Data Sources and Search Strategy

A comprehensive search was performed up to July 2019 with no language restriction, including 8 databases (PubMed, Cochrane Library, EMBASE, Web of Science, China National Knowledge Infrastructure (CNKI), Chongqing VIP (CQVIP), Wanfang Data, and Chinese Biomedical Literature Database (CBM)). Medical subject heading terms (MeSH) and free terms were combined in the process of searching. Moreover, the reference lists of all related articles were examined for relevant citations to ensure the comprehensiveness of the search.

The following key terms and their variations were used in the conduct of a search, including (“Sleep Apnea, Obstructive” OR “sleep apnea” OR “OSA” OR “obstructive sleep apnea” OR “sleep hypopnea” OR “upper airway resistance sleep apnea syndrome” OR “obstructive sleep apnea-hypopnea syndrome” OR “OSAHS” OR “OSAS”) AND (“Acupuncture Therapy” OR “acupuncture” OR “electroacupuncture” OR “manual acupuncture” OR “needling” OR “elongated needling” OR “scalp needle”). Search strategies are presented in [Supplementary-material supplementary-material-1].

### 2.3. Study Selection and Data Extraction

We identified the articles independently according to inclusion and exclusion criteria, first through the title and abstract and afterwards through the full text. And we extracted data from the eligible studies on a standardized data extraction form. Data including author information, publication year, participants, intervention, comparator, and outcomes were extracted. Missing or inconsistent data were managed by contacting authors; if this was not possible, the data would not be included in the meta-analysis. Two authors (Jia Xu and Yijun Zhan) performed the work independently. Disagreement was solved by discussion or a third author (Jian Pei).

### 2.4. Assessment of Risk of Bias

We assessed the risk of bias with the Cochrane Risk of Bias Assessment Tool [16]. Considering the characteristic of acupuncture, it is difficult to blind both the acupuncture operator and patients. Thus, we only focused on whether the assessment of the outcomes was blinded. Two authors (Jia Xu and Yijun Zhan) independently assessed the risk of bias and resolved disputes with the third author (Jian Pei).

### 2.5. Data Synthesis Statistical Analysis

The summary effect size was estimated by using risk ratios (RR) with 95% confidence intervals (CI) for dichotomous outcomes and mean difference (MD) with 95% CI for continuous outcomes. If the same outcome was measured with different methods or scales, we calculated standardized mean difference (SMD). The results were combined in a meta-analysis using RevMan 5.3 software. We applied a fixed effects model to summarize the results when heterogeneity was not relevant (*I*^2^ < 30%). Otherwise, a random effects model was used, since primary RCTs included OSA patients with different severities (mild, moderate, and severe) and this could lead to heterogeneity. Therefore, we separated them into subgroups in all meta-analyses. Sensitivity analysis involving deleting each study separately was carried out in order to assess the quality and consistency of the results and explore the robustness of the findings regarding the study quality and sample size.

### 2.6. Evaluation of the Quality of the Evidence

We evaluated the quality of evidence using GRADE [17] (Grading of Recommendation, Assessment, Development, and Evaluation). We summarized the evidence for each of the outcomes in the table which was filled with the summary of the estimated-risk and 95% confidence intervals. The quality of evidence of each outcome was ranged from high, moderate to low, and very low.

## 3. Results

### 3.1. Literature Search and Study Selection

**Figure 1:**
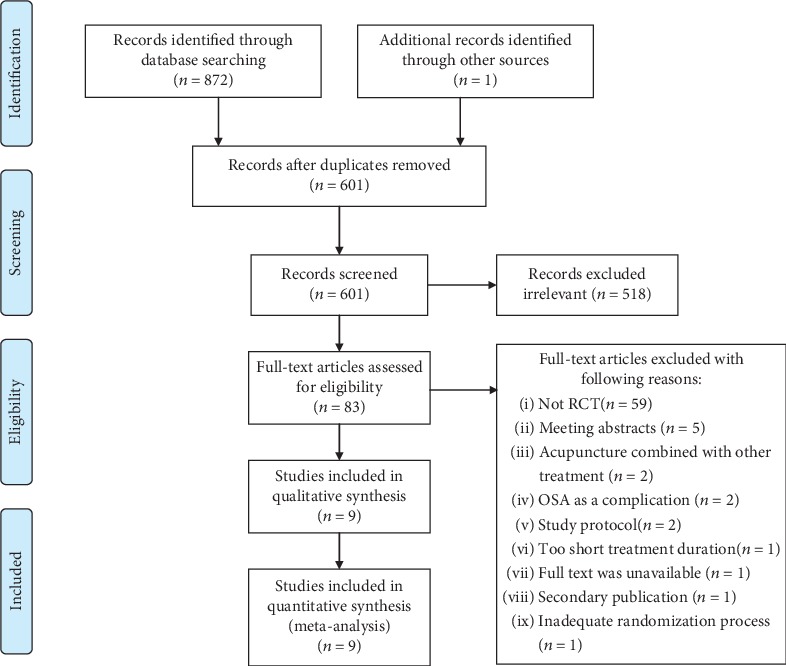
The study selection process.

There were 873 articles in total. One was from a reference list, while the others were identified according to the search strategy. After reviewing the titles and abstracts, 272 duplicated and 518 irrelevant studies were excluded, and 83 potentially relevant studies remained for further assessment. Of these 83 studies, 74 were excluded, leaving 9 eligible randomized controlled trials [12, 18–25]. The study selection process is shown in Figure 1 and the excluded articles are shown in [Supplementary-material supplementary-material-1].

### 3.2. Literature Characteristics

All included studies were RCTs published from 2007 to 2018. One study [12] was conducted in Brazil, and the other eight studies [18–25] were conducted in China. The nine studies included a total of 584 patients, with 304 in the acupuncture group and 280 in the control group. A detailed description of the characteristics of the included studies are shown in Table 1.

### 3.3. Risk of Bias Assessment

**Figure 2:**
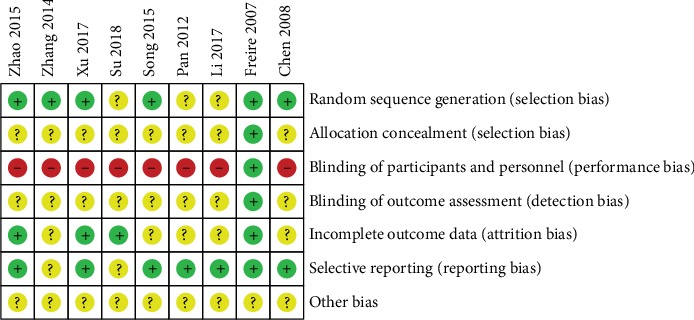
Risk of bias item for each included study.

**Figure 3:**
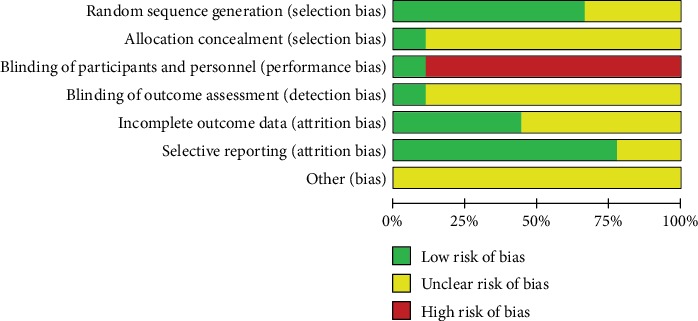
Risk of bias item presented as percentages across all included studies.

Included studies were assessed according to the Cochrane “Risk of Bias” Assessment Tool. Most of the trials are unclear and have a high risk of bias. All of the included studies mentioned randomization. In six studies [12, 18, 21, 23–25], participants were randomly assigned by a random number table. However, three studies [19, 20, 22] failed to report the method of random sequence generation. Only one study [12] reported the methods of allocation concealment, and the blinding of participants and personnel was carried. Patients were unblind in the other eight studies because of the characteristic of intervention. The outcome assessors and statisticians were blinded in one study [12]. Two trials [12, 25] reported dropouts, and three studies [12, 22, 23] reported follow-up. Two studies [22, 24] failed to report outcomes in protocol, and the risk of bias were unclear. All studies did not mention other risks such as factory funding, so the risks of bias were unclear. The details of risk of bias assessment were presented in Figures 2 and 3.

### 3.4. Effects of Interventions

#### 3.4.1. AHI

**Figure 4:**
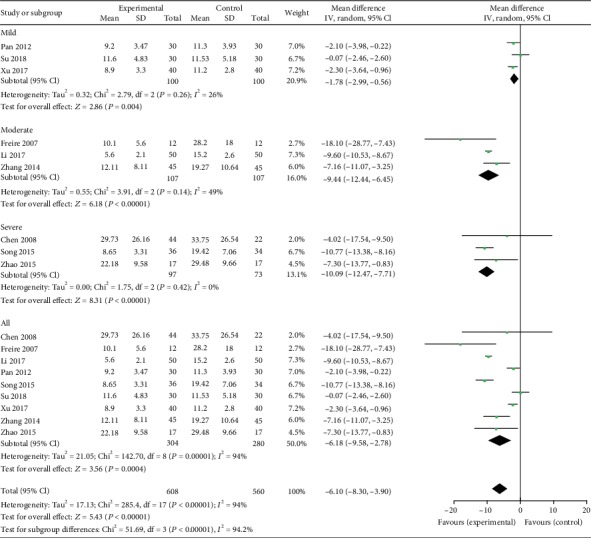
Experimental group versus control group, AHI.

Nine studies [12, 18–25] reported AHI as an outcome. Significant heterogeneity was observed (*χ*^2^ = 285.40, *P* < 0.00001; *I*^2^ = 94%). Therefore, a random-effect model was used. The analysis showed a significant treatment effect on AHI (MD: -6.18; 95% CI: -9.58 to -2.78; *Z* = 3.56*P* = 0.0004). Compared with mild OSA patients (MD: -1.78; 95% CI: -2.99 to -0.56; *Z* = 2.86, *P* = 0.004), AHI decreased more in subgroup analysis in moderate patients (MD: -9.44; 95% CI: -12.44 to -6.45; *Z* = 6.18, *P* < 0.00001) and severe patients (MD: -10.09; 95% CI: -12.47 to -7.71; *Z* = 8.31, *P* < 0.00001) (Figure 4).

#### 3.4.2. ESS

**Figure 5:**
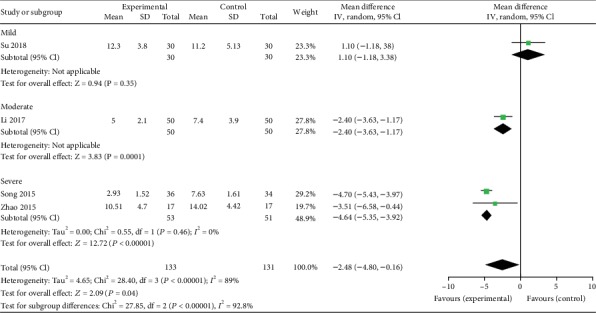
Experimental group versus control group, ESS.

Four studies [19, 21, 22, 25] reported ESS as an outcome. Significant heterogeneity was observed (*χ*^2^ = 28.4, *P* < 0.00001; *I*^2^ = 89%). Therefore, a random-effect model was used. The pooled results showed that ESS in the experimental group decreased more compared to the control group (MD: -2.84; 95% CI: -4.80 to -0.16; *Z* = 2.09, *P* = 0.04). In subgroup analysis, there was no significant decrease in ESS in mild patients (MD: 1.10; 95% CI: -1.18 to 3.38; *Z* = 0.94, *P* = 0.35), while ESS decreased significantly in moderate patients (MD: -2.40; 95% CI: -3.63 to -1.17;*Z* = 3.83, *P* = 0.0001) and severe patients (MD: -4.64; 95% CI: -5.35 to -3.92; *Z* = 12.72, *P* < 0.00001) (Figure 5).

#### 3.4.3. LSaO_2_

**Figure 6:**
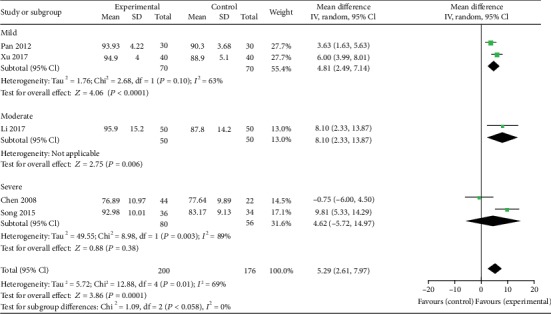
Experimental group versus control group, LSaO_2_.

Five studies [18–21, 23] reported LSaO_2_ as an outcome. Significant heterogeneity was observed (*χ*^2^ = 12.88, *P* = 0.01; *I*^2^ = 69%). Therefore, a random-effect model was used. The pooled results showed that LSaO_2_ in the experimental group improved more compared to the control group (MD: 5.29; 95% CI: 2.61 to 7.97; *Z* = 3.86, *P* = 0.0001). In subgroup analysis, there was no significant decrease in LSaO_2_ in severe OSA patients (MD: 4.62; 95% CI: -5.72 to 14.97; *Z* = 0.88, *P* = 0.38). LSaO_2_ decreased significantly in mild OSA patients (MD: 4.81; 95% CI: 2.49 to 7.14; *Z* = 4.06, *P* < 0.0001) and moderate OSA patients (MD: 8.10; 95% CI: 2.33 to 13.87; *Z* = 2.75, *P* = 0.006) (Figure 6).

### 3.5. Adverse Effect

There were some possible adverse effects of acupuncture, for instance, infection, hematoma, fainting during acupuncture, and bending of needle. In this review, three studies [12, 21, 24] provided information about adverse effects, but no adverse effects were reported about acupuncture.

### 3.6. Publication Biases

We have conducted a comprehensive literature retrieval in the eight databases; conference papers, degree papers, and other documents were also within the scope of retrieval, with no language limitation, in order to minimize the possibility of publication bias. Nevertheless, we could not completely rule out the possibility of publication bias.

### 3.7. Sensitivity Analysis

The data were reanalyzed by deleting each study individually. Most of the outcomes yielded consistent results. However, after excluding the study conducted by Li et al. in 2017 or Song et al. in 2015 or Zhao in 2015 on ESS, the direction of the outcome was reversed (Table 2).

### 3.8. Quality of Evidence

The quality of evidence supporting the main outcome AHI was low. The evidence supporting the efficacy of acupuncture in ESS and LSaO_2_ was very low (Table 3).

## 4. Discussion

### 4.1. Main Findings

This is a further systematic review and meta-analysis to evaluate the efficacy and safety of acupuncture for OSA in various severities. We identified 9 RCTs of acupuncture for OSA, and these studies included 584 patients. With regard to the comparison, our meta-analysis of 9 RCTs showed that acupuncture significantly reduced the AHI of OSA, especially in moderate and severe OSA patients. To our acknowledgement, AHI is used to classify the severity of the disease; people with an AHI value of 5~15, 16~30, or more than 30 times per hour are considered to have mild, moderate, or severe obstructive sleep apnea, respectively [2, 15]. Similarly, AHI is also an important outcome of the efficacy of OSA, but there is no clinically significant threshold of the AHI, and we could not determine the clinical curative effect of acupuncture in the treatment of OSA [1]; however, our meta-analysis results still gave us confidence, since acupuncture could effectively reduce AHI times per hour in OSA and reduce the frequency of sleep-disordered breathing events. Besides, ESS decreased significantly in moderate and severe OSA patients in the acupuncture group, which exceeded the clinical significance threshold (2 points) [1]. Furthermore, the acupuncture group was superior to the control group in improving LSaO_2_. Meanwhile, nine RCTs did not report side effects due to acupuncture intervention. Most of the outcomes yielded consistent results after sensitivity analysis. However, the direction of the outcome of ESS was reversed after excluding the studies, which suggested that the results of ESS was unstable. The quality of evidence evaluated by GRADE in the included studies was mainly low to very low. High-quality trials are urgently needed.

### 4.2. Comparison to Previous Review

The previous review included 6 RCTs for analysis, including a total of 362 OSA patients, and revealed that both manual acupuncture (MA) and electroacupuncture (EA) were effective in improving AHI and mean SaO_2_, and MA could further improve the apnea index and the hypopnea index when compared with the control group; however, no definite conclusion could be drawn due to the limited evidence [13]. Our review did not include two RCTs [26, 27] that were included in the previous review, because one of them provided data on cerebrovascular disease patients [26] and the treatment duration of acupuncture for OSA did not meet the inclusion criteria in the other one [27]. In order to produce robust results, we performed rigorous inclusion criteria and included only RCTs that clearly stated the enrollment of patients specifically referred to OSA. Based on the previous review, we included another 5 RCTs [19, 20, 22, 23, 25], which not only added OSA subjects, but also made our evidence more robust. In addition, our review was aimed at evaluating the effectiveness of acupuncture on three different degrees (mild, moderate, and severe) of OSA, which would be closer to clinical practice, making it easier for physicians to make decisions.

### 4.3. Advantages of Acupuncture Treatment

We recommend acupuncture as an adjuvant therapy for OSA because it has some of the following advantages. Acupuncture has an acute effect in reducing AHI as well as the number of nocturnal respiratory events of OSA patients [27], which can play a therapeutic effect on OSA patients more rapidly. At present, the most effective therapy to reduce OSA is positive airway pressure (PAP), and CPAP provides a constant level of positive pressure across inspiration and expiration [28]. However, it requires tremendous effort on the patient's part to position the mask properly and maintain the machine and supplies. And direct side effects have been reported with the use of PAP, including headache, chest discomfort, and sense of suffocation or difficulty exhaling. These side effects can result in sleep disruption and poor sleep quality, thereby reducing patient adherence to CPAP [29]. Thus, a complementary therapy such as acupuncture may be warranted when treatment intolerance due to side effects occurs. Besides, compared with acupuncture, CPAP has the greater cost-effectiveness, while acupuncture has advantage in cost. Furthermore, all studies included mentioned the arrival of qi when acupuncture was performed and the retention of the needle. The most commonly used acupoints were Zhaohai (KI6), Sanyinjiao (SP6), Sishencong (EX-HN1), Shenmen (HT7), Zusanli (ST36), and lianquan (CV23). Point selection and arrival of qi were essential in the acupuncture treatment for OSA. Acupuncture plays an effective role only if the operating procedure was observed.

### 4.4. Limitations and Weaknesses

There were some limitations in this review. Firstly, acupuncture treatment for OSA has some bias and heterogeneity. The reasons may be the various interventions of acupuncture. Secondly, in this review, OSA patients were categorized as mild, moderate, and severe according to AHI before treatment. AHI is an important measure which is used to diagnose and categorize disease severity of OSA patients. However, there are inherent limitations with using the AHI calculated from one night of sleep to categorize disease severity, because the AHI is influenced by many factors and may vary over time and even across consecutive nights [2]. Thirdly, the methodological quality of the included RCTs was generally low. For example, most of the included studies had a high risk of performance bias.

## 5. Conclusions

Acupuncture may be an effective and safe treatment in OSA patients. Besides, acupuncture may also reduce AHI and ESS and improve LSaO_2_ of OSA patients. This result is more significant in moderate and severe OSA patients. These evidences may be useful to clinicians, patients, and health policy makers with regard to the application of acupuncture in OSA. However, further high-quality RCTs are needed to confirm the efficacy and safety of acupuncture for OSA patients.

## Figures and Tables

**Table 1:** Characteristics of included studies and basic information of included patients.

Author year	Country	No. of patients	Gender (M/F)	Age	BMI	Severity	Intervention		Duration, frequency	Retaining (min)	Outcomes
							T	C			
Freire et al. 2007 [12]	Brazil	24 T 12 C 12	/	/	T 26.9 (26.6-28.0) C 28.6 (24.8-28.8)	Moderate	Acupuncture	Receiving no treatment	10 weeks, 1 per week	30	AHI, adverse effects
Chen et al. 2008 [18]	China	66 T 44 C 22	T 28/16 C 16/6	T 55.44 ± 11.04 C 56.73 ± 10.21	T 26.24 ± 3.58 C 27.11 ± 2.94	Severe	Acupuncture	nCPAP	20 days, 7 per week	30	AHI, LSaO_2,_
Li et al. 2017 [19]	China	100 T 50 C 50	T 29/21 C 27/23	T 50.7 ± 3.5 C 51.1 ± 3.3	/	Moderate	Acupuncture+nCPAP	nCPAP	4 weeks, 7per week	30	AHI, ESS, LSaO_2_
Pan 2012 [20]	China	60 T 30 C 30	T 27/3 C 26/4	T 42.43 ± 10.36 C 41.73 ± 10.12	T 24.91 ± 2.45 C 25.04 ± 2.57	Mild	Electroacupuncture	CPAP	20 days, 7 per week	5	AHI, LSaO_2,_
Song et al. 2015 [21]	China	70 T 36 C 34	T 24/12 C 23/11	T 53.17 ± 10.2 C 52.71 ± 11.26	T 29.05 ± 4.03 C 29.13 ± 3.94	Severe	Electroacupuncture+nCPAP	nCPAP	6 weeks, 3 per week	30	AHI, ESS, LSaO_2_, adverse effects
Su 2018 [22]	China	60 T 30 C 30	T 19/11 C 18/12	T 50.34 (27-70) C 49.5 (28-70)	/	Mild	Acupuncture	nCPAP	4 weeks, 7 per week	30	AHI, ESS
Xu et al. 2017 [23]	China	80 T 40 C 40	T 23/17 C 24/16	T 51.8 ± 5.0 C 49.7 ± 5.4	T 28.8 ± 2.5 C 29.4 ± 2.4	Mild	Electroacupuncture+Chinese medicine	Chinese medicine	8 weeks, 3 per week	30	AHI, LSaO_2_
Zhang et al. 2014 [24]	China	90 T 45 C 45	T 40/5 C 38/7	T 48.45 ± 9.76 C51.96 ± 9.87	/	Moderate	Acupuncture	CPAP	4 weeks,/	30	AHI, adverse effects
Zhao 2015 [25]	China	34 T 17 C 17	T 15/2 C 13/4	T 31.47 ± 6.21 C 32.76 ± 5.96	/	Severe	Acupuncture	Western medicine	4 weeks, 6 per week	20	AHI, ESS

Abbreviations: T—treatment group; C—control group; nCPAP—nasal continuous positive airway pressure; AHI—apnea-hypopnea index; ESS—Epworth Sleepiness Scale; LSaO_2_—lowest oxygen saturation.

**Table 2:** Results of sensitivity analysis.

Outcome	Deletion	Result	
AHI	Chen et al. 2008	*χ* ^2^ = 142.58, *P* < 0.00001, *I*^2^ = 95%	MD-6.28, 95% CI: -9.77 to -2.79
	Freire et al. 2007	*χ* ^2^ = 138.07, *P* < 0.00001, *I*^2^ = 95%	MD-5.42, 95% CI: -8.90 to -1.95
	Li et al. 2017	*χ* ^2^ = 54.56, *P* < 0.00001, *I*^2^ = 87%	MD-5.39, 95% CI: -8.49 to -2.30
	Pan 2012	*χ* ^2^ = 119.72, *P* < 0.00001, *I*^2^ = 94%	MD-6.84, 95% CI: -10.59 to -3.10
	Song et al. 2015	*χ* ^2^ = 131.27, *P* < 0.00001, *I*^2^ = 95%	MD-5.50, 95% CI: -9.20 to -1.79
	Su. 2018	*χ* ^2^ = 115.86, *P* < 0.00001, *I*^2^ = 94%	MD-7.10, 95% CI: -10.62 to -3.58
	Xu et al. 2017	*χ* ^2^ = 95.82, *P* < 0.00001, *I*^2^ = 93%	MD-6.82, 95% CI: -10.55 to -3.09
	Zhang et al. 2014	*χ* ^2^ = 142.56, *P* < 0.00001, *I*^2^ = 95%	MD-6.07, 95% CI: -9.78 to -2.35
	Zhao 2015	*χ* ^2^ = 142.63, *P* < 0.00001, *I*^2^ = 95%	MD-6.07, 95% CI: -9.68 to -2.46

ESS	Li et al. 2017	*χ* ^2^ = 22.60, *P* < 0.0001, *I*^2^ = 91%	MD-2.43, 95% CI: -6.24 to 1.38
	Song et al. 2015	*χ* ^2^ = 8.30, *P* = 0.02, *I*^2^ = 76%	MD-1.55, 95% CI: -4.05 to 0.94
	Su 2018	*χ* ^2^ = 10.06, *P* = 0.007, *I*^2^ = 80%	MD-3.60, 95% CI: -5.40 to -1.80
	Zhao 2015	*χ* ^2^ = 28.38, *P* < 0.00001, *I*^2^ = 93%	MD-2.19, 95% CI: -4.97 to 0.58

LSaO_2_	Chen et al. 2008	*χ* ^2^ = 7.90, *P* = 0.05, *I*^2^ = 62%	MD-6.15, 95% CI: 3.67 to 8.62
	Li et al. 2017	*χ* ^2^ = 11.75, *P* = 0.008, *I*^2^ = 74%	MD-4.86, 95% CI: 1.89 to 7.83
	Pan 2012	*χ* ^2^ = 9.63, *P* = 0.02, *I*^2^ = 69%	MD-5.89, 95% CI: 2.18 to 9.61
	Song et al. 2015	*χ* ^2^ = 8.17, *P* = 0.04, *I*^2^ = 63%	MD-4.39, 95% CI: 1.80 to 6.99
	Xu et al. 2017	*χ* ^2^ = 11.43, *P* = 0.010, *I*^2^ = 74%	MD-5.10, 95% CI: 1.06 to 9.15

**Table 3:** Quality of evidence evaluated by grading of recommendation assessment, development, and evaluation.

Outcome	Number of studies	Sample size	Confidence intervals (95%)	GRADE					Evidence quality
				Limitation	Inconsistency	Indirections	Imprecision	Publication bias	
AHI	9	584	MD: -6.18; 95% CI: -9.58 to -2.78	-1	-1	0	0	0	Low
ESS	4	264	MD: -2.84; 95% CI: -4.80 to -0.16	-1	-1	0	-1	0	Very low
LSaO_2_	5	376	MD: 5.29; 95% CI: 2.61 to 7.97	-1	-1	0	-1	0	Very low

## Supplementary Materials

Supplementary Materials Appendix 1 shows the details of the literature search strategy. Appendix 2 shows the basic information of the excluded articles.
