# Supplementary material for: Acupuncture for Obstructive Sleep Apnea (OSA) in Adults: A Systematic Review and Meta-Analysis
Source: Biomed Res Int. 2020 Mar 5;2020:6972327. doi: 10.1155/2020/6972327 (PMC7079261; doi:10.1155/2020/6972327)
Supplement: Supplementary Materials — Appendix 1 shows the details of the literature search strategy. Appendix 2 shows the basic information of the excluded articles. [file 6972327.f1.zip › appendix/Appendix 1 Search strategy.pdf]

Appendix I

Details of the Literature Search Strategy

(1) PubMed （1977 to July 7, 2019）

Search	Query	Items found
#1	"Sleep Apnea, Obstructive"[Mesh]	18916
#2	((((((((Sleep Apnea, Obstructive [Title/Abstract]) OR sleep apnea [Title/Abstract]) OR OSA [Title/Abstract]) OR obstructive sleep apnea [Title/Abstract]) OR upper airway resistance sleep apnea syndrome [Title/Abstract]) OR obstructive sleep apnea-hypopnea syndrome [Title/Abstract]) OR OSAHS [Title/Abstract]) OR OSAS [Title/Abstract])	42393
#3	#1OR#2	45364
#4	("acupuncture"[Mesh]) OR ("Acupuncture Therapy"[Mesh])	23697
#5	(((((((Acupuncture Therapy [Title/Abstract]) OR acupuncture [Title/Abstract]) OR electroacupuncture [Title/Abstract]) OR manual acupuncture [Title/Abstract]) OR needling [Title/Abstract]) OR elongated needling [Title/Abstract]) OR scalp needle [Title/Abstract])	24123
#6	#4 OR #5	30457
#7	#3 AND #6	25

(2) Embase (1974 to July 7, 2019)

Search	Query	Items found
#1	'Sleep disordered breathing'/exp	73280
#2	'Sleep Apnea, Obstructive':ab,ti OR 'sleep apnea':ab,ti OR OSA:ab,ti OR 'obstructive sleep apnea':ab,ti OR 'sleep hypopnea':ab,ti OR 'upper airway resistance sleep apnea syndrome':ab,ti OR 'obstructive sleep apnea-hypopnea syndrome':ab,ti OR OSAHS:ab,ti OR OSAS:ab,ti	58903
#3	#1 OR #2	81770
#4	'acupuncture'/exp	44981
#5	'Acupuncture Therapy':ab,ti OR Acupuncture:ab,ti OR Electroacupuncture :ab,ti OR 'manual acupuncture':ab,ti OR needling:ab,ti OR 'elongated needling':ab,ti OR 'scalp needle':ab,ti	35327
#6	#4 OR #5	48662
#7	#3 AND #6	104

(3) Cochrane Library (July 7, 2019)

Search	Query	Items found
#1	MeSH descriptor: [Sleep Apnea, Obstructive] explode all trees	1609
#2	(“Sleep Apnea, Obstructive”):ti,ab,kw OR (“sleep apnea”):ti,ab,kw OR (OSA):ti,ab,kw OR (“obstructive sleep apnea”):ti,ab,kw OR (“sleep hypopnea”):ti,ab,kw	5949
#3	(“upper airway resistance sleep apnea syndrome”):ti,ab,kw OR (“obstructive sleep apnea-hypopnea syndrome”):ti,ab,kw OR (“OSAHS”):ti,ab,kw OR (“OSAS”):ti,ab,kw	3722
#4	#1 OR #2 OR #3	6611
#5	MeSH descriptor: [Acupuncture] explode all trees	141
#6	(“Acupuncture Therapy”):ti,ab,kw OR (Acupuncture):ti,ab,kw OR (Electroacupuncture):ti,ab,kw OR (“manual acupuncture”):ti,ab,kw OR (“needling”):ti,ab,kw	14775
#7	(“elongated needling”):ti,ab,kw OR (“scalp needle”)	2046
#8	#5 OR #6 OR #7	15569
#9	#4 AND #8	19

(4) Web of Science core collection (1986 to July 7, 2019)

Search	Query	Items found
#1	Topic: (Sleep Apnea, Obstructive) OR Topic: (sleep apnea) OR Topic: (OSA) OR Topic: (obstructive sleep apnea) OR Topic: (sleep hypopnea) OR Topic: (upper airway resistance sleep apnea syndrome) OR Topic: (obstructive sleep apnea-hypopnea syndrome) OR Topic: (OSAHS) OR Topic: (OSAS)	49619
#2	Topic: (Acupuncture Therapy) OR Topic: (Acupuncture) OR Topic: (Electroacupuncture) OR Topic: (manual acupuncture) OR Topic: (needling) OR Topic: (elongated needling) OR Topic: (scalp needle)	110198
#3	#1 AND #2	86

(5) Chinses database

<table>
<tr><th>database</th><th>Query</th><th>Items found</th></tr>
<tr><td>CNKI</td><td rowspan="4">主题:(OSAHS+睡眠呼吸暂停综合征+睡眠呼吸暂停，阻塞性+睡眠呼吸暂停+睡眠呼吸障碍+睡眠呼吸疾病+睡眠呼吸紊乱+阻塞性睡眠呼吸暂停低通气综合征+睡眠呼吸暂停低通气综合征+鼾症)*主题:(针灸+针刺+电针)</td><td>94</td></tr>
<tr><td>WANFANG DATA</td><td>164</td></tr>
<tr><td>VIP</td><td>100</td></tr>
<tr><td>Sinomed</td><td>279</td></tr>
</table>
