# Supplementary material for: Acupuncture for Obstructive Sleep Apnea (OSA) in Adults: A Systematic Review and Meta-Analysis
Source: Biomed Res Int. 2020 Mar 5;2020:6972327. doi: 10.1155/2020/6972327 (PMC7079261; doi:10.1155/2020/6972327)
Supplement: Supplementary Materials — Appendix 1 shows the details of the literature search strategy. Appendix 2 shows the basic information of the excluded articles. [file 6972327.f1.zip › appendix/Appendix 2 Basic information of the excluded articles.pdf]

Appendix 2: Basic information of the excluded articles

First author	Year	Title	Reason for exclusion
Buhebayaer, Q.	2012	Treatment of moderate obstructive sleep apnea syndrome with acupuncture: A randomized, placebo-controlled pilot trial	Meeting abstract
Thornton, T.	2015	The effect of acupuncture on upper airway physiology in obstructive sleep apnoea	Meeting abstract
Thornton, T.	2015	The effect of acupuncture on upper airway function and physiology and subjective outcomes in obstructive sleep apnoea	Meeting abstract
Freire, A.	2007	Immediate effect of acupuncture on the sleep architecture of patients with obstructive sleep apnea	Meeting abstract
Freire, A.	2010	Research conducted in a federal university, Sao Paulo, Brazil related to the treatment of obstructive sleep apnoea with acupuncture	Meeting abstract
Peili Zhang	2014	Electroacupuncture treatment of 50 cases of obstructive sleep apnea hypopnea syndrome	OSA as a complication
Peili Zhang	2014	Treating 30 patients with obstructive sleep apnea hypopnea syndrome by laryngeal three acupoints	OSA as a complication
Barlas, P.	2011	Acupuncture may help with sleep apnoea	Study protocol
Bijak, M.	2007	Treatment of moderate obstructive sleep apnea syndrome with acupuncture: a randomised, placebo-controlled pilot trial	Study protocol
Weiguo Chen	2009	Clinical study on acupuncture and sublingual	Acupuncture com-

		acupuncture for treatment of obstructive sleep apnea hypopnea syndrome	bined other treatment
Dan Zhang	2017	Clinical observation of local electroacupuncture combined with pharyngeal cavity and genioglossus muscle to improve nighttime ventilation in patients with OSAHS	Acupuncture combined other treatment
Xin Zheng	2018	Protective effect of Chinese medicine combined with acupuncture on obstructive sleep apnea hypopnea syndrome: A randomized controlled study	Inadequate randomization process
Freire, A. O.	2010	Immediate effect of acupuncture on the sleep pattern of patients with obstructive sleep apnoea	Too short treatment duration
Ruilong Liang	2010	Clinical study of acupuncture on sleep-disorder regulation in patients with obstructive sleep apnea hypopnea syndrome	Full text was unavailable
Xiaofan Yu	2014	The clinical observation and mechanism analysis of the xuan fei jian pi acupuncture therapy for the phlegm-dampness type OSAHS	Secondary publication
